# Sphingolipid metabolites involved in the pathogenesis of atherosclerosis: perspectives on sphingolipids in atherosclerosis

**DOI:** 10.1186/s11658-024-00679-2

**Published:** 2025-02-07

**Authors:** Fufangyu Zhao, Mingyan Shao, Mingrui Li, Tianxing Li, Yanfei Zheng, Wenlong Sun, Cheng Ni, Lingru Li

**Affiliations:** 1 National Institute of Traditional Chinese Medicine Constitution and Preventive Medicine, Beijing University of Chinese Medicine, Beijing, 100029 China; 2 School of Traditional Chinese Medicine, Beijing University of Chinese Medicine, Beijing, 102488 China; 3 Institute of Biomedical Research, School of Life Sciences, Shandong University of Technology, Zibo, 255000 Shandong China

**Keywords:** Sphingolipids, Phytosphingolipids, Atherosclerosis, Metabolites, Enzymes

## Abstract

Atherosclerosis, with its complex pathogenesis, is a leading underlying cause of many cardiovascular diseases, which are increasingly prevalent in the population. Sphingolipids play an important role in the development of atherosclerosis. Key metabolites and enzymes in sphingolipid metabolism influence the pathogenesis of atherosclerosis in a variety of ways, including inflammatory responses and oxidative stress. Thus, an investigation of sphingolipid metabolism-related metabolites and key enzymes may provide novel insights and treatment targets for atherosclerosis. This review discusses various mechanisms and research progress on the relationship between various sphingolipid metabolites, related enzymes, and atherosclerosis. Finally, we look into the future research direction of phytosphingolipids.

**Graphical Abstract:**

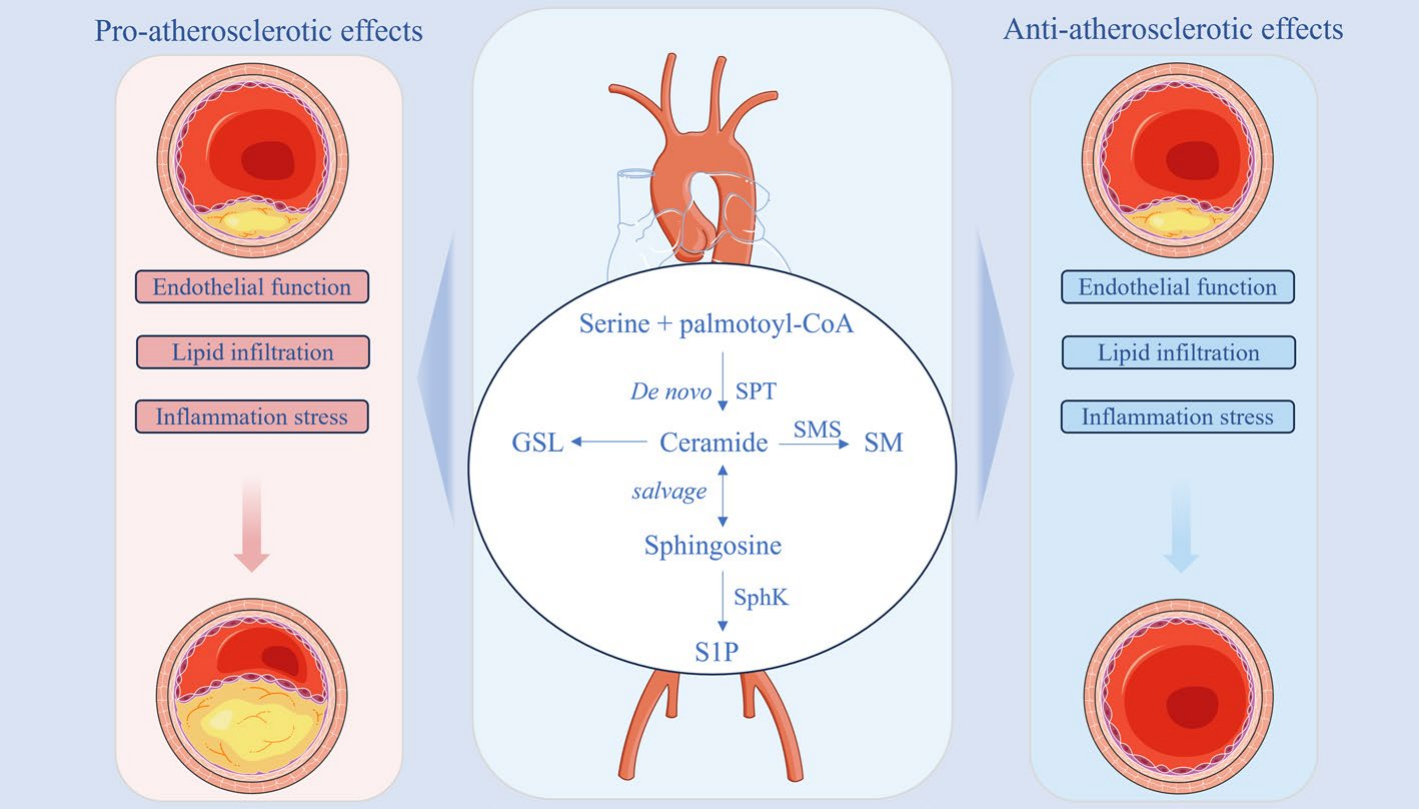

## Introduction

Atherosclerosis, the underlying cause of the majority of cardiovascular diseases, has emerged as a significant global public health concern [1]. Currently, the incidence of atherosclerotic cardiovascular disease remains high in China. In the absence of a family history and related diseases, the likelihood of a 60-year-old Chinese individual developing atherosclerosis within 10 years stands at 11.0% for men and 10.1% for women, with the incidence still increasing with age [2]. Through animal experiments including rabbit, pig, primate, and rodent models, modern scholars have confirmed that lipid metabolism, endothelial damage, and immune inflammation play roles in the development of atherosclerosis and its complex mechanisms [1, 3–8]. Currently treatments for atherosclerosis include statins, PCSK9 inhibitors, and antiplatelet and anti-inflammatory agents [9]. However, while statins continue to demonstrate their therapeutic benefits, they also potentially affect cardiac and vascular muscle function [10]. PCSK9 inhibitors are a relatively new type of lipid-lowering drug [11] that prolongs the life span of low density lipoprotein receptor (LDLR) and maintains cholesterol homeostasis in vivo, making them innovative pharmacological targets for the treatment of atherosclerosis [12]. Such drugs still have the potential to lead to the occurrence of allergic symptoms.

In the nineteenth century, Thudicum’s team first extracted an amphiphilic membrane lipid from brain tissue and named it sphingolipid [13]. The potential link between sphingolipids and atherosclerosis was first described by Smith in 1960 when she found increased levels of sphingolipids in atherosclerotic vascular lesions [14]. In recent decades, research have discovered that sphingolipid metabolism is involved in atherosclerosis from its formation to the development of secondary lesions, transforming sphingolipid metabolism and sphingolipid-related molecules into biomarkers and therapeutic targets for cardiovascular diseases [15–17]. Sphingolipids have been shown to have specific effects on cellular processes involved in the development of atherosclerosis, such as influencing nitric oxide (NO) production [18–20], apoptosis [21–24], low-density lipoprotein (LDL) aggregation [21, 25, 26], and inflammatory processes [27]. Meanwhile, as a complex signaling network, the various sphingolipids involved during sphingolipid metabolism can complement and constrain each other, thus preventing the rapid development of atherosclerosis that might be due to the imbalance of a particular sphingolipid [28].

Different subunit compositions also result in various physiological roles for sphingolipids. For example, serine palmitoyltransferase (SPT) consists of three larger subunits, Sptlc1, Sptlc2, and Sptlc3, and one of two smaller subunits, ssSPTa or ssSPTb [29–32]. Sptlc1 and Sptlc2 are universally expressed in mammals, whereas Sptlc3 is only expressed in certain tissues, such as the placenta, skin, and some glands [33]. Sptlc1 and Sptlc2 are primarily involved in the formation of long-chain sphingosine bases such as C18, C19, and C20, whereas SPT complexes containing Sptlc3 lead to the production of C16 sphingomyelin bases [33]. Additionally, Sptlc3 is predominantly associated with the exercise of sphingosine bases as well as the formation of anteiso-branched-C18 SO (meC18SO) [29, 33]. MeC18SO is a component of human LDL and high-density lipoprotein (HDL) and can be metabolized into ceramide (CER) and complex sphingolipids [29]. Owing to the presence of different subunits, sphingolipids may perform distinct physiological functions and may even exert bidirectional regulation of disease. In the following section, we describe the metabolic processes and structure of sphingolipids and the involvement of various sphingolipid metabolites in atherosclerosis. We also discuss the potential role of phytosphingolipids in atherosclerosis.

### Structure and function of sphingolipids

**Fig. 1:**
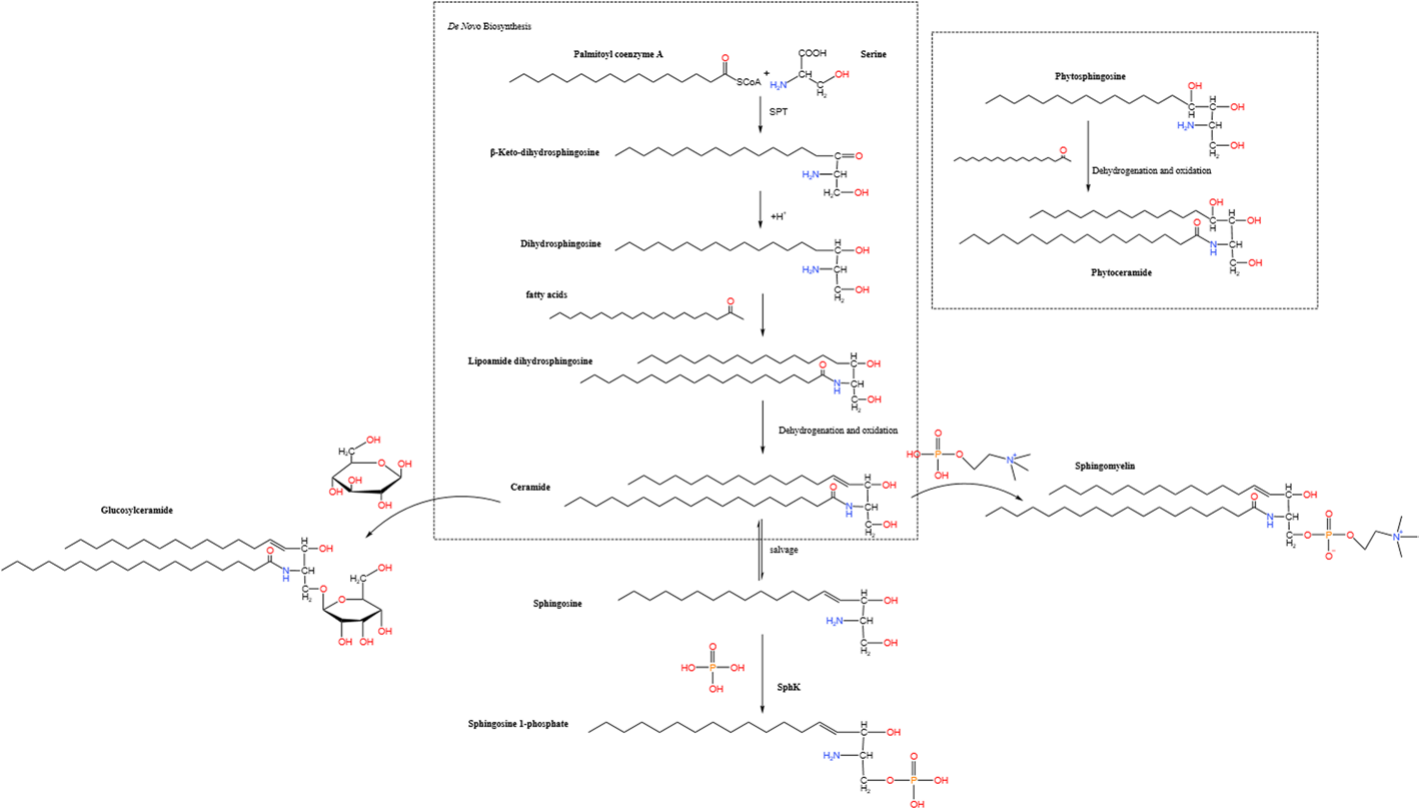
De novo biosynthesis and the structure of sphingolipids and their derivatives

Sphingolipids, a class of amphiphilic lipids, are composed of a sphingoid backbone with a long-chain fatty acid at one end and a polar alcohol structure at the other [17]. Sphingosine is the simplest member of the sphingolipid family and can be interconverted with ceramides. Ceramides are intermediate metabolites of sphingolipids, which are a class of amide compounds formed by the dehydration of long-chain fatty acids with the amino groups. Sphingosine-1-phosphate is another derivative, formed by the combination of sphingosine with a molecule of phosphate. Additionally, ceramide can be phosphorylated by a choline phosphate group to form sphingomyelin. Sphingolipids encompass sphingomyelins, sphingosines, and glycosphingolipids, among others [13]. Glucosylceramide, the simplest known glycosphingolipid found in plants, contains a glucose moiety (Fig. 1) [34, 35].

The various sphingolipids mentioned above undergo mutual transformation, a process catalyzed by enzymes during sphingolipid metabolism within the body. Under physiological conditions, the mutual transformation of sphingolipids helps maintain the homeostasis of sphingolipid levels in the human body. Sphingolipids play a diverse range of roles, encompassing several aspects such as cell growth, apoptosis, cellular senescence, cell adhesion and autophagy, inflammation, immunity, angiogenesis, metabolism, nutrient uptake, etc. [13, 36, 37].

In the de novo synthesis pathway, palmitoyl coenzyme A and serine combine to form β-keto-dihydrosphingosine in the presence of SPT. The carbonyl group is then hydrogenated and reduced, yielding dihydrosphingosine. The amino group of dihydrosphingosine condenses with a fatty acid to form lipoamide-based dihydrosphingosine. Ultimately, it undergoes dehydrogenation and oxidation to form ceramide. In addition to the interconversion between ceramides and sphingosine, these molecules can combine various groups to generate derivatives and sphingolipids.

### Sphingolipids biosynthesis and metabolism

Sphingolipids inherently maintain homeostatic turnover [38]. The accumulation of sphingolipids in the human body begins with the synthesis of ceramides (CERs) [39]. CERs are pivotal in sphingolipid metabolism [17]. Three identified pathways are responsible for their synthesis [40]: (1) De novo biosynthesis: As shown in Fig. 1, this process occurs in endoplasmic reticulum (ER). Serine and palmitoyl coenzyme A undergo a key reaction catalyzed by SPT to produce 3-ketosphinganine, which is then converted to dihydrosphingosine by 3-ketosphinganine reductase (KSR) [41–43]. Sphingosine is subsequently modified by N-acylation through the catalytic activity of ceramide synthase, leading to the formation of dihydroceramide. Further modification occurs via dihydroceramide desaturase, which introduces a *trans*-4,5 double bond, ultimately yielding CER [38, 40, 44]. Nearly all eukaryotic cells produce CERs primarily through the de novo synthesis pathway [39]. (2) Sphingomyelinase (SMase) pathway: This process takes place in several organelles such as the endoplasmic reticulum/Golgi complex and lysosomes. Sphingomyelin (SM) is catalyzed by acidic SMase or neutral SMase to produce CERs [45]. (3) Salvage pathway: Also known as the sphingolipid cycle pathway, this process typically occurs in lysosomes or endosomes. Multiple complex sphingolipids are converted to CER by enzymes and then to Sph by ceramidase. Sph is recycled back to the ER, where it is catalyzed by ceramide synthase to regenerate CER. At least half of the sphingosine is reutilized through this pathway, which plays an important role in maintaining sphingolipid homeostasis [38, 46].

**Fig. 2:**
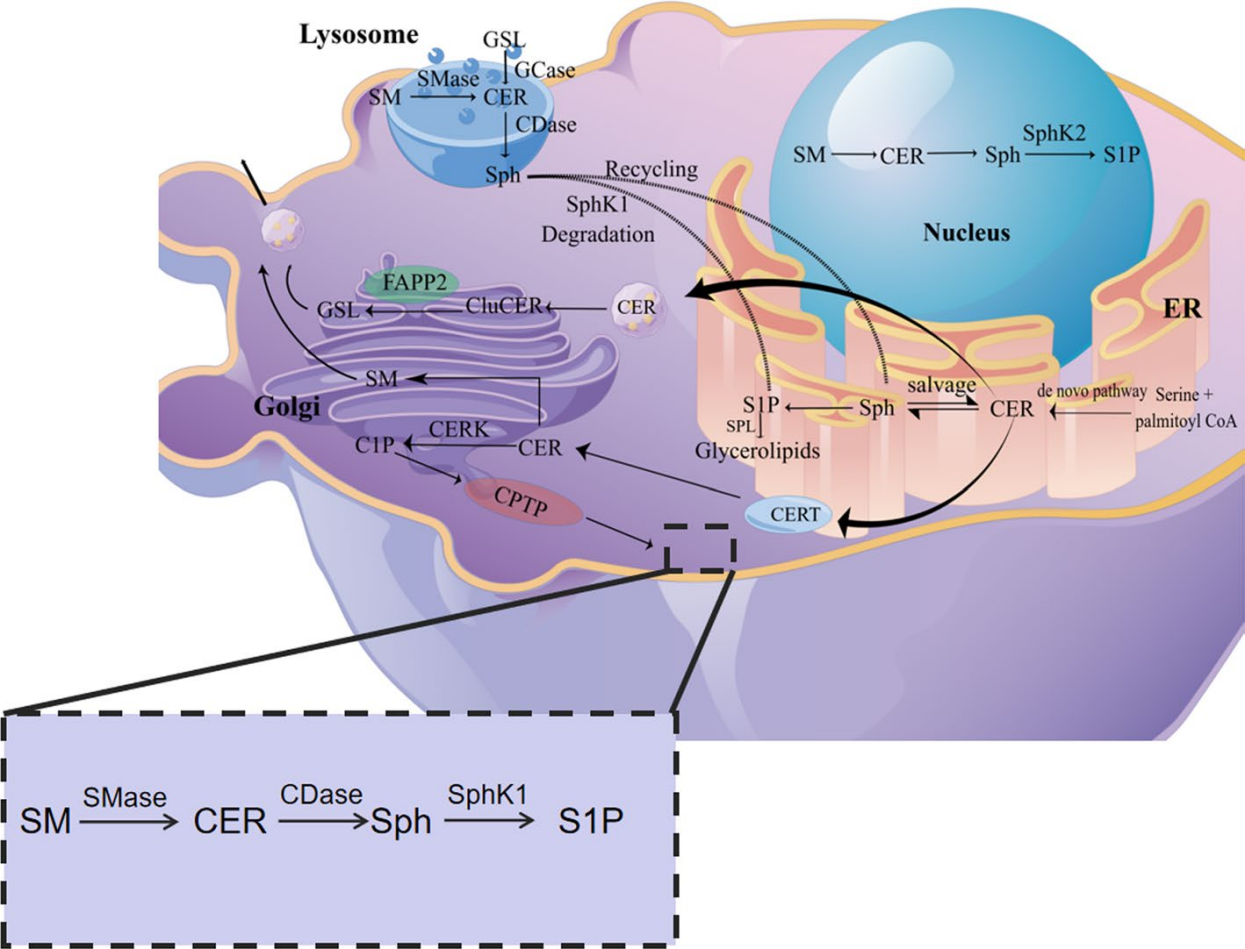
Sphingolipid metabolism

As a prerequisite and basic structure for complex sphingolipids, CER can be transformed into several sphingolipid types. In the ER, ceramidase deacetylates CER to form sphingosine, which is then phosphorylated by sphingosine kinase to form sphingosine-1-phosphate (S1P) [39]. Sphingosine phosphate lyase, the final enzyme in the sphingolipid degradative pathway, catalyzes the irreversible cleavage of long-chain base phosphates, yielding hexadecenal (a long-chain aldehyde) and phosphoethanolamine. Ceramide transport proteins (CERT) facilitate transport CER from the ER to the Golgi apparatus, where it is phosphorylated by phosphorylcholine moiety, and catalyzed by sphingomyelin synthase to produce SM [38, 47]. In the Golgi, CER can also be phosphorylated by ceramide kinase (CERK) to produce ceramide-1-phosphate (C1P), a rare sphingolipid that serves as an important signaling molecule. C1P promotes cell growth and proliferation, maintains vascular and epithelial integrity, and plays a modulatory role in inflammation and cancer [38, 48]. In addition to CERT, CER can also be transported from the ER to the Golgi via vesicles and synthesized into glucosylceramide (GluCER). Four-phosphate adaptor protein 2 (FAPP2) transports GluCER to the trans-Golgi to form glycosphingolipids (GSL). Glycosphingolipids have a more complex structure [38, 49]. Both GSL and SM can be translocated to the cell membrane via vesicular transport, while C1P is translocated to the cell membrane by C1P-specific transfer protein (CPTP). Summarily, the roles of various sphingolipids produced by CER metabolism vary depending on their molecular composition and spatial structure [13, 49, 50]. Figure 2 shows the process of sphingolipid metabolism.

In the ER, serine and palmitoyl CoA undergo the de novo synthesis pathway to synthesize ceramide (CER) and synthesize sphingosine (Sph). Sph can synthesize CER through the salvage pathway and further synthesize sphingosine-1-phosphate (S1P) catalyzed by sphingosine kinase (SphK) (probably SphK1). CER can be transported to the Golgi apparatus by ceramide transport protein (CERT) to synthesize sphingomyelin (SM). Meanwhile, CER could synthesize ceramide-1-phosphate (C1P) catalyzed by ceramide kinase. C1P could be transported to the cell membrane with the help of C1P-specific transfer protein (CPTP). CER can also be transported via vesicles to the Golgi apparatus for the synthesis of glucosylceramide (GluCER). Four-phosphate adaptor protein 2 (FAPP2) transports GluCER to the trans-Golgi and then synthesizes complex glycosphingolipids (GSLs). Both GSLs and SMs can be translocated to the cytosol by vesicular transport. In lysosomes, both GSLs and SMs are involved in the synthesis of CERs, which is facilitated by the presence of glycosidase (GCase) and SMase (SMase). CERs can then be transformed into sphingosine (Sph) through the catalysis of ceramidase (CDase). The Sph can undergo recycling by SphK1 to generate sphingosine-1-phosphate (S1P), which can further participate in the synthesis of glycerolipids with the help of S1P lyase (SPL). Alternatively, Sph can also enter the salvage pathway. The synthesis process from SM to S1P can also occur in the cytosol. On the other hand, the process in the nucleus is primarily catalyzed by SphK2.

### The roles of sphingolipids in atherosclerosis

In the early stages of atherosclerosis, cholesterol-laden macrophages accumulate beneath the endothelium, forming “fatty streak” lesions. Subsequently, plaque formation, luminal surface calcification, ulceration, and bleeding of small vessels within the vessel wall into the lesion occur, along with vascular thrombosis. These processes ultimately lead to thickening and hardening of the arterial wall and narrowing of the lumen [3]. As shown in Table 1, sphingolipids play an important role in the progression of atherosclerosis [28, 51, 52]. Metabolites and key enzymes involved in sphingolipid metabolism can exert either a positive or a negative influence on atherosclerosis, impacting its course at every stage. Below, we detail the effects of sphingolipid metabolism on atherosclerosis, considering both metabolites and key enzymes in this metabolic pathway.

**Table 1:** Association of key metabolites and enzymes in sphingolipid metabolism with endothelial function, lipid lamination, and inflammatory responses during atherosclerosis

Metabolites and enzymes	Endothelial function	Lipid infiltration	Inflammation stress
CER	Increased ROS production	Formation of LDL aggregates	Promotion of MMP-7 secretion
	Reduction of NO production by PP2A	Promotion of LDL oxidation	Induction of IL-6, IL-10, and MCP-1 release
	Release of granulosa cytochrome C or activation of caspase-8 promotes endothelial cell apoptosis		
SM		Retention of cholesterol in cells and artery walls	
		Subendothelial aggregation and retention of atherogenic lipoproteins	
S1P	Binding to S1PR affects endothelial cell integrity	biased distribution on HDL	Elevated local S1P levels can cause increased levels of endothelial adhesion molecules
	Attenuating endothelial cell injury by activating the AKT/eNOS signaling pathway	Levels are influenced by the metabolism of ApoM lipoproteins	Recruitment of inflammatory cells
	Damage to the endothelial barrier (high glucose and high concentration conditions)		Activation of dendritic cells
GSL	Promoting cellular phagocytosis of endothelial cells	Accumulation with Ox-LDL in fatty streaks and endothelial plaques	
	Accumulates in the vascular endothelium and promotes thrombosis		
SPT	Regulation of vascular endothelial function and proliferation	Regulation of cholesterol efflux	Regulation of the inflammatory response in macrophages
SMS	Promoting endothelial dysfunction by activating the Wnt/β-catenin signaling pathway	Regulation of macrophage phagocytosis of lipids to form foam cells	Targeting SM levels on macrophage membranes and lipid rafts to regulate the NF-kappaB pathway
			Upregulation of inflammatory biomarker expression
SphK		Involvement in ABCA1-mediated regulation of cholesterol efflux	Promotes vascular inflammation
		Maintenance of autophagic degradation of lipid droplets in macrophages	Promotes expression of endothelial cell adhesion molecules

### Sphingolipids as biomarkers in atherosclerosis identification and progression

Studies have reported a significant accumulation of CERs in individuals with atherosclerosis and obesity [53]. Specific types of CERs have been identified as predictive biomarkers for future adverse cardiovascular events [54]. Plasma CERs are recommended as biomarkers for predicting cardiovascular events, and determination of plasma CER species and their ratios predicts the risk of major acute cardiovascular events and death [55]. In a prospective study of 1704 Chinese patients with coronary artery disease, followed up for an average of 9 years, plasma CER C16:0, C18:0, and C24:1 were found to be strongly associated with increased cardiovascular events and all-cause mortality. After multivariate adjustment, a one-standard-deviation increase in the ratios of CER C16:0/C24:0, C18:0/C24:0 ratio, and C24:1/C24:0 ratio was associated with a 27%, 35%, and 21% increase in the risk of death from cardiovascular events, respectively, and a 29%, 28%, and 24% increase in the risk of all-cause death, respectively. Patients with higher CER risk scores had a 1.81-fold and 1.95-fold increased risk of death from cardiovascular events and all-cause mortality, respectively, compared with those with lower scores. Meanwhile, in patients with atherosclerotic cardiovascular disease, plasma CER levels may have higher predictive value than conventional biomarkers for predicting the risk of cardiovascular event onset and death [56–58]. Serum CERs can also predict the risk of cardiovascular disease in healthy individuals [59, 60].

Plasma SM levels were first assessed using a high-throughput enzymatic assay by Jiang et al. They discovered that plasma SM is an independent risk factor for coronary heart disease (CHD) and is present at high levels in the CHD population [61]. Additionally, higher SM levels were found in atherosclerotic lesion tissues compared with normal vessels [62]. Increased carotid intima–media thickness, a subclinical manifestation of early atherosclerosis, has been shown to be positively correlated with plasma SM levels [63]. However, a 5-year cohort study evaluating the predictive value of plasma SM for cardiovascular disease found that SM levels were not associated with an increased risk of developing coronary heart disease [64]. Therefore, it is not entirely certain that elevated plasma SM levels indicate an increased risk of atherosclerosis development. S1P has also received extensive attention in cardiovascular disease studies. Plasma S1P has been found to have a higher predictive accuracy than HDL in atherosclerosis and is negatively associated with atherosclerotic diseases such as peripheral arterial disease and carotid artery stenosis [65]. As complex lipids with biological activity, sphingolipids may serve as biomarkers for atherosclerosis and partially predict the risk of atherosclerosis development. Circulating blood levels of CER and SM may be the more definitive risk factors in cardiovascular disease, while most studies have shown a protective effect of S1P against cardiovascular disease [66]. We now discuss in detail the mechanisms of various sphingolipids in atherosclerosis.

### Mechanisms linking CERs to atherosclerosis

CERs modulate three pathological aspects of atherosclerosis development. In endothelial cells, CERs contribute to endothelial dysfunction through various mechanisms, such as increasing the production of reactive oxygen species (ROS) and decreasing NO production via the activation of protein phosphatase 2A (PP2A) [15]. CERs act as a second messenger, triggering the activation of the cAMP-dependent protein kinase (CAPK), Ras-associated factor-1 (Raf-1), extracellular regulated protein kinases (ERK) cascade, stress-activated protein kinase/c-Jun N-terminal kinase (SAPK/JNK) cascade, nuclear factor kappa-B (NF-κB) activation, and dead cell receptor aggregation. These events, in turn, can lead to the release of mitochondrial cytochrome C or the activation of caspase-8, promoting the apoptosis of endothelial cells [67]. The detrimental roles of CERs in oxidative stress and apoptosis within endothelial cells can disrupt endothelial homeostasis and exacerbate atherosclerosis development [51]. From microscopic observations, it is clear that CERs can be a key factor in atherosclerosis at the cellular level. However, cellular alterations are just one aspect of the development of atherosclerosis, and the in vivo development is often more complex.

The accumulation of LDL beneath the endothelium is essential for the formation of plaque during the initial stages of atherosclerosis. CERs accumulate in lipoprotein particles, destabilizing the particles, which in turn causes a conformational change in ApoB100, promoting the formation of larger LDL aggregates, which are more conducive to fatty streak formation [68]. Additionally, CER-containing LDL aggregates are engulfed by macrophages, leading to the secretion of matrix metalloproteinase 7 (MMP-7) [68], which is involved in foam cell formation and inflammatory activation, significantly accumulating at the fatty streak. Both inflammatory responses and foam cells may reduce the stability of atherosclerotic plaques and increase the risk of complications [69, 70]. Meanwhile, CER-LDL induces the release of IL-6, IL-10, and monocyte chemotactic protein 1(MCP-1) through CD14 and TLR4 activation in monocytes [71].

As atherosclerosis progresses, fatty streaks gradually evolve into fibrous plaques. CERs are enriched in human coronary atherosclerotic plaques and accumulate predominantly in macrophages near the necrotic core [72]. Owing to local inflammation of the plaque, cells within the plaque can also undergo apoptosis or even necrosis, thereby increasing the risk of rupture of unstable plaque [73]. Deep tissue necrosis and disintegration in fibrous plaques, owing to malnutrition, further aggravate vascular obstruction and stenosis. A large amount of ceramide exists in atheromatous plaques [74]. CER also participates in the progression of atherogenesis and atherosclerotic plaque formation by promoting the oxidation of LDL in vascular smooth muscle cells [52]. In secondary lesions of atherosclerosis, plasma CER is significantly associated with plaque rupture [75] and mediates the cytosolic action of Weibel–Palad bodies to cause thrombosis [76]. Aggregation of CERs has also been found in calcified plaques [77]. In summary, plasma CER is closely associated with the onset, development, and formation of atherosclerosis and equally implicated in the secondary lesions of the disease, making it an important factor in disease progression.

Nonetheless, CER may protect the vascular endothelium during the early stages of atherosclerosis. Studies have confirmed that the de novo synthesis of endothelial sphingolipids is an important source of CERs in plasma and endothelial cells require de novo CER synthesis to maintain vascular tone and blood pressure homeostasis [78]. In terms of cell-mediated vasodilatory function, C16:0-Cer plays a major role in maintaining tyrosine kinase and G-protein coupled receptors-induced vasodilation, whereas blood flow-mediated vasodilation is majorly regulated by C24:0-Cer and C24:1-Cer [78]. NO is a crucial endothelium-derived relaxing factor that mediates cardiovascular protection and anti-atherosclerotic function [79]. De novo synthesis of endothelial sphingolipids responds to NO signaling and suppresses eNOS phosphorylation primarily through ceramide to control blood pressure.

Owing to variations in the carbon chain length, degree of unsaturation, and number of hydroxyl groups in both the fatty acid portion and sphingosine portion of ceramides, the types of ceramides are quite diverse. Over 300 types of ceramides have been identified to date. Consequently, CERs show diverse effects in atherosclerosis in the human body [80]. Long-chain ceramides (such as 24:0) are mostly considered benign metabolites, whereas short-chain ceramides (such as 16:0) can exacerbate obesity and insulin resistance [81–83]. Different structures also correspond to different metabolites and different physiological functions [84]. Common mammalian ceramides are metabolized to sphingosine, which is metabolized to even-chain fatty acids. In contrast, phytoceramides are metabolized to phytosphingosine, which features an additional hydroxyl group and is metabolized into odd-chain fatty acids [85, 86]. The level of odd-chain fatty acids is negatively associated with the risk of cardiovascular disease development [87], suggesting that phytoceramides can improve cardiovascular disease through their unique metabolic characteristics.

### Mechanisms linking SM to atherosclerosis

SM is the major sphingolipid in mammalian cell membranes and is mostly synthesized in the trans-Golgi apparatus and in the extracellular leaflets of the plasma membrane [88]. SM synthesis was also found in the nucleus [89]. Plasma levels of SM are regarded as an independent risk factor for atherogenicity [90]. Elevated levels of lipoproteins and SM in the arterial wall were found in an animal model of diet-induced atherosclerosis, which may be the result of dietary induction leading to activation of SPT in aortic cells [91]. In addition, disruption of the SPT gene in the mouse liver limited the synthesis of long-chain sphingomyelin bases in their livers, resulting in reduced SM levels in the liver membrane and plasma [92].

SM causes may contribute to atherosclerosis, possibly owing to its ability to retain cholesterol within cells and artery walls. Sphingomyelin synthase (SMS) catalyzes the synthesis of SM. Overexpression of SMS1 and SMS2 in mice can lead to an increased probability of the development of atherosclerosis [93]. Further studies have shown that plasma level of SM, total cholesterol, triglycerides, and LDL-cholesterol levels in mice increased with SMS2 activation [94]. Inhibition of de novo SM biosynthesis can reduce plasma cholesterol and triglyceride levels, increases HDL cholesterol levels, and prevents the development of atherosclerosis [95–97]. Additionally, Ca^2+^ deposition in the arterial wall has also been found to be associated with SM content in atherosclerotic tissue, which can be related to electrostatic interactions between Ca^2+^ and SM [98, 99].

The aggregation and retention of atherogenic lipoproteins beneath the endothelium are key to the formation of atherosclerosis [100, 101]. SM is present in all major lipoproteins, particularly in atherogenic ones such as very low density lipoprotein (VLDL) and LDL [102, 103]. SM appears to play a significant role in the subendothelial aggregation and retention of atherogenic lipoproteins. SMase in the arterial wall promotes the aggregation and retention of SM-rich LDL in atherosclerotic lesions, initiating the early stages of atherosclerosis development [53]. It has been suggested that selective reduction of SM content in atherogenic lipoproteins inhibits their retention in mouse aorta and subsequent development of atherosclerosis [104].

The mechanisms by which SM in atherosclerosis leads to the aggregation and retention of lipoproteins involve multiple biological processes. Initially, SM can modify lipoproteins, especially LDL, leading to changes in their function that increase retention in the arterial wall. For instance, oxidative modification of LDL increases its interaction with the arterial wall and promotes the development of atherosclerosis [53]. Oxidized low-density lipoprotein (ox-LDL) is thought to be involved in atherogenesis, and ox-LDL may contain oxysterols [105, 106]. It has been demonstrated that some of the oxysterols can promote SM production, which leads to SM accumulation in the arterial wall [107]. Additionally, SMase in the arterial wall hydrolyzes SM to produce CERs, which accumulate to increase the permeability of the vessel wall and promote lipoprotein permeation and retention [44]. Sphingomyelin has been identified as an important component of lipid rafts, and the formation of these rafts enhances the interaction of lipoproteins with cellular receptors, leading to endocytosis and lipoprotein retention [108]. Meanwhile, SM can interact with extracellular matrix (ECM) components, such as binding to elastin and collagen fibers, and this interaction can promote retention of lipoproteins in the arterial wall [109]. Metabolites of SM can activate an inflammatory response that attracts immune cells such as macrophages to the arterial wall, and these cells can phagocytose modified lipoproteins, forming foam cells that further promote atherosclerotic plaque formation [110]. Some researchers have also noted that the presence of sphingolipids can affect the stability of lipoprotein particles, making them more prone to aggregate. For example, LDL particles containing sphingomyelin may be more likely to form large aggregates that are more easily captured by the arterial wall [68]. Lipoproteins aggregated and retained in the arterial wall undergo oxidative modification (ox-LDL) and can cause inflammatory responses and damage to endothelial cells, promoting the development of atherosclerosis [111, 112]. Lipoprotein aggregation and retention also stimulate the proliferation and migration of vascular smooth muscle cells (SMCs), which are involved in plaque formation and stabilization [113].

### Mechanisms linking S1P to atherosclerosis

S1P serves as a signaling lipid and is predominantly found in the circulatory system, including blood and lymphatic vessels. S1P is present at lower concentrations within tissues [38]. Approximately two-thirds of S1P in plasma is carried by high-density lipoproteins, with albumin being the next most significant carrier [114]. Apolipoprotein M (ApoM), a less abundant protein found on HDL particles, acts as a transporter of S1P molecules within HDL structure [115]. S1P can be transported by apoM/HDL or albumin, promoting its interaction with receptors on the cell surface and exerting pleiotropic properties [116]. Recent studies have shown that S1P possesses both pro-atherosclerotic and anti-atherosclerotic properties [114]. Understanding the factors underlying this phenomenon might help explain the dual role of S1P in atherosclerosis. Reports indicate that the properties of apoM/HDL-bound S1P differ from those of albumin-bound S1P, which may at least partially explain the dual nature of S1P in the fields of atherosclerotic [116].

Local S1P levels rise in the inflammatory state, triggering endothelial adhesion molecules, recruiting inflammatory cells, and activating dendritic cells. Meanwhile, S1P protects the endothelium by preventing endothelial cell–cell interactions, reducing vascular permeability, and suppressing cytokine-induced leukocyte adhesion [117]. S1P also activates AKT/eNOS signaling pathway to mitigate endothelial cell injury induced by intermittent hypoxia [118]. Several studies have shown that S1P also exerts pro-atherosclerotic properties. Besides the aforementioned sections, S1P features are also influenced by its concentration. Lower concentrations (around 0.1 mmol/L) of S1P play an important role in maintaining endothelial barrier integrity. However, increased concentrations (approximately 10 mmol/L) of S1P can be detrimental to the endothelial barrier, causing an increase in vascular permeability [119]. S1P stimulation in HCAECs reduced NO level and eNOS activity, while simultaneously increasing polymorphonuclear neutrophil (PMN) adherence and the expression of adhesion molecules, including VCAM-1 and ICAM-1 under both high-glucose and normal conditions. These findings suggest that S1P causes endothelial cell dysfunction [120]. In atherosclerosis and inflammation, researchers have proposed that S1P exhibits chemotactic properties toward macrophages, with their migration toward S1P relying on the presence of the S1P3 receptor in macrophages [121]. Several studies indicate that S1P can induce only a weak platelet shape change response, increase in cytosolic Ca^2+^, and subsequent platelet aggregation. Notably, platelet shape change acts as the initial response of platelets, occurring before aggregation and adhesion [122]. In summary, S1P and its receptors are key players in the atherosclerotic disease process. Although S1P and its receptors exhibit a dual nature in atherosclerosis, a comprehensive understanding of its properties will help in developing novel pharmacological approaches to atherosclerosis based on S1P agonists and antagonists.

S1P binds to a variety of sphingosine-1-phosphate receptors (S1PR1–5), influencing the development of atherosclerosis in terms of vascular tone, endothelial cell function and integrity, among other factors [117, 123]. S1PR1, S1PR2, and S1PR3 are mainly expressed in cardiovascular tissues, S1PR4 is mainly expressed in the lymphatic system, while S1PR5 is characteristic of the immune and nervous systems [124]. Research findings indicate that, in high-glucose conditions, S1PR1 expression in human coronary artery endothelial cells (HCAECs) was significantly downregulated, whereas that of S1PR2 underwent a significant upregulation. S1PR1 overexpression or S1PR2 knockdown could alleviate oxidative stress and the dysfunction of HUVECs [125]. Activation of S1PR1 protects LDL receptor knock-out mice from atherosclerosis [126]. In addition, knockdown of the S1PR2 gene in apoE-deficient mice under conditions of reduced macrophage recruitment in vivo attenuated their atherosclerotic plaques [127]. Recent studies have shown that the deletion of endothelial cells (ECs)-S1PR2 causes improved post-ischemic angiogenesis and blood flow recovery in ischemic tissues. This mechanism involved in suppressing the AKT/eNOS signaling pathway has several aspects, including cell growth, apoptosis, cellular senescence endothelial cell proliferation, migration, and angiogenic activity [128]. It has also been shown that S1PR3 knock-out mice exhibit resistance to the protective properties of HDL and S1P in coronary infarction modeling experiments, while carotid artery ligation in mice knocked out for the S1PR3 gene leads to an increase in macrophage recruitment and neointima formation [121, 129]. S1P exerts a protective effect on endothelial cells through S1PR1/3, binds to S1PR2 to inhibit smooth muscle cell migration, and has also been shown to exert an anti-inflammatory effect through S1PR4 [114, 130]. There are few studies on the mechanistic link between S1PR5 and atherosclerosis, but some studies have suggested that S1PR5 could be a potential noninvasive screening biomarker for coronary heart disease [131]. In conclusion, the binding of S1P to different receptors can have different effects on the development of atherosclerosis; for example, S1PR1 and S1PR3 primarily exert anti-atherosclerotic effects, while S1PR2 mainly exerts pro-atherosclerotic effects. This also explains the bidirectional regulation of atherosclerosis development by S1P as mentioned above.

### Mechanisms linking GSL to atherosclerosis

Glycosphingolipid (GSL) is an important product of sphingolipid metabolism and a universal component of the eukaryotic plasma membrane, comprising a ceramide backbone linked to a glycan component [132]. GSLs are the most complex type of sphingolipid. Depending on the order and type of sugar residues attached to the head group, GSLs form dozens of different sphingolipids. The different structures also correspond to different functions [133].

Some GSLs closely associated with lipid rafts can transport gut flora metabolites from the plasma membrane to the endoplasmic reticulum and through the Golgi apparatus into the cytoplasm for them to function. For instance, a GSL on endothelial cells called globotriaosylceramide (Gb3Cer/CD77) possesses an oligosaccharide component in its structure that can be identified by (and bind to) Shiga toxin (Stx) produced by *Escherichia coli* (STEC), which promotes endothelial cell cytophagy by Stx [134, 135]. Lactosylceramide (LacCer), a GSL found in vascular cells, comprises a lactosyl and ceramide backbone linkage [136]. LacCer accumulates in fatty streaks and endothelial plaques along with a plaque-specific lipoprotein called oxidized low-density lipoprotein (Ox-LDL) [137] and promotes atherosclerosis development in conjunction with growth factors and pro-inflammatory cytokines [77]. Meanwhile, structural differences may also cause differences in GSL aggregation sites. One study found high glucosylceramide, trihexosylceramide, and tetrahexosylceramide in the elastic layer cells of the lipo-striated muscle [138]. Unlike in other sites, ganglioside G_M3_ aggregates more in the intimal hyperplastic layer of the atherosclerotic aortic wall [139]. Notably, thrombosis is an important secondary lesion in the atherosclerotic process. Globotriaosylceramide (Gb3) is a major GSL accumulating in the vascular endothelium. Excess endothelial Gb3 accumulation causes increased thrombosis, atherosclerosis, and endothelial dysfunction [140]. Moreover, large amounts of LacCer accumulation have been found in calcified atheromatous plaques [77]. Therefore, GSL plays a role in atherosclerotic plaque calcification, and its structure is connected to its site of action.

### Enzymatic involvement in the metabolism of sphingolipids in atherosclerosis development

#### The role of SPT and its inhibitors in the progression of atherosclerosis

SPT is a key rate-limiting enzyme in sphingolipid metabolism, catalyzing the first step in sphingolipid metabolism: the condensation of palmitoyl coenzyme A and l-serine. During experiments using the CRISPR/Cas9 method to generate Sptlc1 and Sptlc2 knockout HAP1 cell lines, it was found that Sptlc2 was lost with the loss of Sptlc1, whereas deletion of the Sptlc2 gene did not have an effect on the level of Sptlc1, suggesting that Sptlc2 is unstable in the absence of Sptlc1, and that Sptlc1 is inherently stable [29]. SPT has been shown to influence the development of atherosclerosis by regulating macrophage inflammatory response and cholesterol efflux. This process may be related to the reduction of macrophage membrane SM mediated by Sptlc2 deficiency [141]. Meanwhile, another study indicated that Sptlc1 is required for vascular development and systemic sphingolipid homeostasis [142]. This suggests that SPT not only affects macrophage inflammatory responses and cholesterol efflux but also plays a regulatory role in vascular endothelial function and proliferation. Recent study has shown that, although Sptlc3 does not bind directly to electron transport chain (ETC) complex I, there is a novel interaction between Sptlc3-derived LacCer and complex I [143]. Sptlc3 deficiency leads to a decrease in the activity of complex I, which in turn reduces cardiomyocyte adenosine triphosphate (ATP) and reactive oxygen species (ROS) production [143]. SPTLC3 variants have also been noted to be associated with elevated low-density lipoprotein cholesterol, myocardial infarction, and dyslipidemia [144–146].

Sptclt1 and Sptclt2/Sptclt3 serve as larger subunits that together form the catalytic core of SPT. In addition to the larger subunit, a third smaller subunit, ssSPTa or ssSPTb, can improve catalytic efficiency while providing substrate specificity for fatty acyl-CoA substrates [147]. There are few studies on the mechanisms associated between ssSPT and atherosclerosis, but it has been shown that ssSPT can increase SPT activity without affecting substrate specificity [148]. This suggests that the smaller moiety has the ability to indirectly modulate atherosclerosis, which is achieved by affecting the activity of the SPT.

Myriocin, a natural fungal product, has been identified to be the most potent inhibitor of SPT. Research table myriocin can ameliorate atherosclerosis in ApoE−/− mice by reducing lipid uptake and vascular inflammation [149]. The specific pathway may be the downregulation of MCP-1 and its receptor chemokine receptor 2 (CCR2) expression as well as cluster of differentiation 36 (CD36) and lectin-like ox-LDL receptor-1 (LOX-1) expression. Myriocin reduces endogenous ceramide and significantly inhibits transendothelial transcytosis of ox-LDL [150]. Meanwhile, myriocin also reduces plasma SM and cholesterol levels [151]. This shows that myriocin, as an SPT-specific inhibitor, ameliorates atherosclerosis in a variety of ways, both by affecting vascular inflammation and by reducing lipid uptake as well as cholesterol levels. On the other hand, treatment with myriocin on a high-fat diet resulted in a decrease in plasma sphingolipid levels, with no significant changes in total cholesterol and triglycerides, but a significant reduction in atherosclerotic lesion area, which also suggests that SPT has a pro-atherosclerotic character. Therefore, inhibition of SPT activity may be a potential alternative for the treatment of atherosclerosis [152]. The enzymatic activity of SPT is regulated by three orosomucoid-like proteins (ORMDL1–3). These three proteins form a complex with SPT and negatively regulate SPT function [153]. For example, ORMDL3 is located at the center of the SPT-ORMDL complex and interacts with the subunits of SPT through hydrogen bonding and salt bridges, etc., thus stabilizing the conformer and blocking the binding between SPT and the substrate at the same time [154–156]. In the absence of one ORMDL isoform, the remaining two isoforms have been shown to compensate for this deficiency and maintain the inhibitory effect on SPT [157]. Currently, there are relatively few studies on the mechanisms associated between ORMDL and atherosclerosis. One study suggests that ORMDL3 increases the risk of atherosclerosis in the Chinese Han population [158]. In this study, shRNA-mediated silencing of ORMDL3 alleviated ox-LDL, basal, and serum starvation-induced endothelial cell autophagy to a certain extent, suggesting that ORMDL3 may be a causative gene associated with endothelial cell autophagy in the pathogenesis of atherosclerosis.

#### The roles of sphingomyelin synthases in the progression of atherosclerosis

Sphingomyelin synthases (SMS), key factors in the sphingolipid synthesis pathway, are a class of membrane proteins that catalyze CER as one of the substrates for the production of SM [159]. Three types of SMS have been identified, including SMS1, SMS2, and SMS-related protein (SMSr). Both SMS1 and SMS2 synthesize sphingomyelin, with SMS1 being located primarily in the Golgi apparatus, whereas SMS2 is more prevalent in the cell membrane. SMSr plays an important role in catalyzing the synthesis of the SM analog ceramide phosphoethanolamine (CPE) in the ER lumen [160].

SMSs regulate the occurrence of atherosclerosis. At the stage of endothelial injury, SMS2 promotes endothelial dysfunction by activating the Wnt/β-catenin signaling pathway [161]. Macrophages play a critical role in atherosclerosis occurrence and are an important cell type in atherosclerotic lesion formation [162]. After the endothelial injury, foam cell accumulation formed by macrophages that phagocytose huge amounts of fat results in fatty streaks. Researchers have proposed that the involvement of macrophages in atherogenesis may be regulated by sphingomyelins distributed across the cell membrane [141, 163]. Sphingomyelin levels on macrophage membranes are strongly linked to cholesterol utilization and inflammatory responses. Lipid molecules including sphingolipids, cholesterol, and glycolipids interact with each other to form specialized structures known as lipid rafts in the cell membrane [164]. This structure can be involved in cell signaling, lipid and protein transport, as well as maintenance of membrane structure. NF-kappaB has long been recognized as a proatherogenic factor. SMS2 can regulate NF-kappaB activation by targeting the SM levels on macrophage membranes and lipid rafts, thereby regulating NF-kappaB activation [165]. In the atheromatous plaque stage, SMS2 overexpression significantly upregulates the expression of aortic matrix metalloproteinase-2 (MMP-2), monocyte (MCP-1, tissue factor (TF), and cyclooxygenase-2 (COX-2) biomarkers of aortitis. On the other hand, SMS2 overexpression decreases circulating levels of CD34/KDR-positive cells, circulating angiogenic cells (CACs), and colony forming units (CFUs), thereby increasing atheromatous plaque instability [94, 166]. SMS2 is a positive regulator of platelet activation and thrombosis, and participates in thrombus formation in secondary lesions of atherosclerosis [167].

Because of their role in atherosclerosis, SMSs may be potential therapeutic targets [17]. In vivo experiments found that SMS2 gene knockout in ApoE-deficient mice resulted in a reduction in inflammatory response and atherosclerotic lesions [104]. Moreover, SMS1 deletion in mice reduced sphingomyelin levels in plasma, liver, and macrophages. This inhibited TLR4-mediated activation of NF-κB and MAP kinase on macrophage membrane lipid rafts, and attenuated inflammatory responses, resulting in anti-atherosclerotic effects [95]. Macrophage SMS1-deficient mice exhibit anti-atherosclerotic effects similar to that of SMS2-deficient mice in a mouse model [95]. However, they also display various severe functional abnormalities including insulin secretion defects [168], CD4+ T-cell dysfunction [169], dysfunction of lipid storage in adipocytes [170], sperm developmental defect [171], and mesenchymal transformation of the epithelium in the renal papillary collecting ducts [172].

#### The roles of sphingosine kinases in the progression of atherosclerosis

Sphingosine kinases (SphKs) primarily participate in S1P production as part of sphingolipid metabolism. Furthermore, SphKs play a crucial role in regulating CER and SM levels, hence a central enzyme in regulating these lipid levels. SphKs are of two types, i.e., SphK1 and SphK2, among which the latter is majorly found in the nucleus. Being important enzymes involved in sphingolipid metabolism, SphKs participate in atherosclerosis development.

Furthermore, SphK1 modulates cytokine production and expression of adhesive factors in HUVEC cells. SphK1 deficiency abrogated cellular MCP-1 production when microvascular endothelial cells from Sphk1-deficient mice were treated with a protease-activated receptor 1-activating peptide [173], suggesting that SPHK1 inhibition is a novel therapeutic approach for atherosclerosis. These pieces of evidence suggest that SphK1 promotes vascular inflammation and expression of endothelial cell adhesion molecules, which are closely associated with atherosclerosis preformation.

SphK1 and SphK2 have distinct, isoform-specific roles in atherosclerosis [174]. Recent research demonstrated the specific involvement of SphK2 in the regulation of cholesterol efflux mediated by ABCA1 [175]. Research findings indicate that SphK2 plays a protective role in autophagy, hence suppressing cholesterol accumulation in macrophages. In Western diet (WD)-fed Sphk1−/− mice and Sphk2−/− mice, both with an ApoE-deficient background, the exacerbation of atherosclerosis in mice lacking Sphk2, but not Sphk1, was attributed to impaired autophagic degradation of lipid droplets (LDs) in macrophages [174].

In LDL-R-deficient mice, the inhibition of Sphks causes both pro-atherogenic consequences (via enhanced activation of dendritic cells and T-cells) and anti-atherogenic effects (via reduced activation of endothelial cells). Researchers have speculated that BC294640 targets SphK1 and SphK2 at different concentrations; nonetheless, it is possible that both isoforms were not equally suppressed during treatment, thereby explaining the bidirectional regulatory effect of SphKs on atherosclerosis [176]. Research on the mechanism of SphKs remains incomplete, indicating a need for further investigation including the relationship between SphK1 and SphK2.

### Participation of sphingolipids produced by symbiotic microbiota in host metabolic processes

Serum metabolite profiles and gut microbiota of CAD patients are markedly different compared with healthy controls, suggesting that metabolites and gut microbiota may be further disrupted during CAD [177]. The human metabolome comprises endogenous metabolites, exogenous metabolites, intestinal microbial metabolites, and bacterial–host cometabolites. The human gut microbiota extensively interacts with the host via metabolic exchange and substrate cometabolism [178], and sphingolipids are no exception. Sphingolipid levels negatively correlate with the degree of atherosclerosis and cardiac markers in intestinal flora metabolites of the CAD population [177]. Therefore, sphingolipids produced by intestinal bacteria can enter the host metabolic pathways and influence ceramide levels [178], in turn influencing endothelial cell function and atherosclerosis.

### Symbiotic microbiota regulates sphingolipid metabolism and may be a promising approach for anti-atherosclerotic interventions

We already know that sphingolipids produced by intestinal flora can participate in physiological processes within the host and improve human health. Focusing on the metabolism of these specific sphingolipids from the symbiotic microbiota will facilitate the study of the gut flora as a new target for the treatment of diseases. Researchers have also begun to focus on sphingolipid biosynthesis and glycosylation modifications within the symbiotic microbiota. It has been reported that alpha-galactosylceramides produced by glycosylated sphingosine and ceramide from the human symbiont *Bacteroides fragilis* (BfaGCs) regulates immune function in humans [179, 180]. Interestingly, α-glucosyl-diacylglycerol (aGlcDAG) produced by gut microorganisms represented by *Enterococcus bgsB* exhibits the opposite lipid structure-specific effect to modulate host immune responses by antagonizing BfaGC-mediated activation of NKT cells [181]. In addition to regulating host immune function, hepatic metabolism is also regulated by intestinal microbial-derived sphingolipids, such as the high serine sphingolipids produced by *Bacteroides thetaiotaomicron*, which can enter the body and improve oxidative respiration and lipid accumulation in hepatocytes [182]. During sphingolipid metabolism in plants and some bacteria, a special sphingolipid is formed known as phytosphingolipid [183, 184]. Phytosphingolipids are less abundant in mammalian cells [185]. Unlike sphingosine, phytosphingosine has no *trans* double bond formation, but a molecule of the hydroxyl group is attached at the 4-carbon position owing to hydroxylation [186]. Functional studies of genes related to phytosphingolipid metabolism indicate that phytosphingolipids play an important role in plant growth, development, and stress response [187]. Also in plant cells, phytosphingolipids can interact with free phytosterols, thereby increasing the level of plasma membrane ordering [188]. However, the effects of phytosphingolipids on the human body are not known yet. It has been found that inhibition of dihydroceramide desaturase 1 (DES1) effectively inhibits the formation of double bonds in sphingomyelin and ceramide structures, thereby reducing lipid accumulation [189]. Therefore, we can speculate that phytosphingolipids can have a protective effect on the human body. However the functional differences between plant sphingolipids and mammalian sphingolipids have not been completely characterized, and more evidence is still needed to support this conjecture.

## Conclusions

Traces of sphingolipid metabolites are detectable throughout the atherosclerotic process. Relevant metabolites and related enzymes in sphingolipid metabolism are closely related to endothelial cell proliferation, senescence, apoptosis, and oxidative stress. Meanwhile, sphingolipid metabolites and their receptors regulate the atherosclerotic process and cardiovascular function. However, atherosclerosis regulation by sphingolipid metabolites may be bidirectional, which is associated with sphingolipid metabolite levels in the body and the tissues and organs in which they are found.

Symbiotic microbiota exerts a significant effect on the human internal environment. Meanwhile, sphingolipid metabolites of symbiotic microbiota can enter the host and participate in the pathways of the host. Thus, intestinal flora is assumed to be involved in host sphingolipid metabolism, in turn influencing the course of atherosclerosis in the host. Furthermore, there is a process of sphingolipid metabolism in the commensal microbiota that can regulate host health by forming specific sphingolipids through their own unique biosynthesis and glycosylation modifications. The sphingolipids formed by this process are diverse, not only in terms of changes in carbon chain groups but also in terms of spatial structure, bond energy, and structural stability, which needs to be further explored by modern scholars.

Phytosphingolipids are predominantly found in plants and some bacteria. In plant plasma membranes, phytosphingolipids can be present in amounts of 30–50%, depending on the organ and tissue in which they are found [188]. Currently, studies have confirmed the positive effects of phytosphingosine and its derivatives in the fight against cancer cells [190], anti-inflammation [191, 192], and the treatment of skin diseases [193, 194], while it has also been shown that phytosphingosine has effects such as induction of apoptosis [195, 196] and inhibition of cell proliferation [197]. The above results suggest that phytosphingolipids may have a bidirectional regulatory role, as do sphingolipids. However, phytosphingolipid may function differently from sphingolipids owing to their stable structural properties. A comprehensive study of the role and regulatory mechanisms of sphingolipids and phytosphingolipids will improve the understanding of the role of phytosphingolipids while exploring new targets and ideas for the treatment of lipid metabolic diseases and cardiovascular-related diseases.

## Data Availability

Not applicable.

## Abbreviations

CER: Ceramides
ER: Endoplasmic reticulum
S1P: Sphingosine-1-phosphate
KSR: 3-Ketosphinganine reductase
CERT: Ceramide transport protein
SM: Sphingomyelin
CERK: Cerimide kinase
C1P: Ceramide-1-phosphate
GluCER: Glucosylceramide
FAPP2: Four-phosphate adaptor protein 2
GSL: Glycosphingolipids
CPTP: C1P-specific transfer protein
Sph: Sphingosine
SphK: Sphingosine kinases
GCase: Glycosidase
SMase: Sphingomyelinase
CDase: Ceramidase
SPL: Sphingosine-1-phosphate lyase
CHD: Coronary heart disease
ROS: Reactive oxygen species
PP2A: Protein phosphatase 2A
CAPK: CAMP-dependent protein kinase
Raf-1: Ras-associated factor-1
ERK: Extracellular regulated protein kinases
SAPK: Stress-activated protein kinase
JNK: C-Jun N-terminal kinase
NF-κB: Nuclear factor kappa-B
MMP-7: Matrix metalloproteinase 7
ApoM: Apolipoprotein M
S1PR: Sphingosine-1-phosphate receptors
HCAECs: Human coronary artery endothelial cells
PMN: Polymorphonuclear neutrophils
Gb3Cer: Globotriaosylceramide
LacCer: Lactosylceramide
Ox-LDL: Oxidized low-density lipoprotein
Gb3: Globotriaosylceramide
SPT: Serine palmitoyltransferase
meC18SO: Anteiso-branched-C18 SO
LOX-1: Ox-LDL receptor-1
ORMDLs: Orosomucoid-like proteins
SMSs: Sphingomyelin synthases
SMSr: SMS-related protein
CPE: Ceramide phosphoethanolamine
MMP-2: Matrix metalloproteinase-2
MCP-1: Monocyte chemotactic protein 1
VLDL: Very low density lipoprotein
TF: Tissue factor
COX-2: Cyclooxygenase-2
CACs: Circulating angiogenic cells
CFUs: Colony forming units
BfaGCs: *Bacteroides fragilis*
aGlcDAG: α-Glucosyl-diacylglycerol
DES1: Dihydroceramide desaturase 1
